# Correlation between the Dendritic Structure and Lattice Parameter of γ′-Phase in Single-Crystalline Turbine Blades Made of Superalloys

**DOI:** 10.3390/ma15030781

**Published:** 2022-01-20

**Authors:** Jacek Krawczyk, Włodzimierz Bogdanowicz

**Affiliations:** Institute of Materials Engineering, University of Silesia in Katowice, 1a 75 Pułku Piechoty St., 41-500 Chorzów, Poland; wlodzimierz.bogdanowicz@us.edu.pl

**Keywords:** nickel-based superalloy, X-ray diffraction, lattice parameter, dendritic array

## Abstract

The dendritic structure and the distribution of the γ′-phase lattice parameter (a_γ′_) along selected lines of the longitudinal section in a model single-crystalline blade made of CMSX-4^®^ nickel-based superalloy were studied. It was established that there is a correlation between the value of the a_γ′_ and the predomination of initial or ending fragments of the secondary dendrite arms. It is most noticed for the areas where the dendrite growth conditions are similar to steady. They are located in the center and near the root’s selector extension (SE) area. The correlation has been related to the dendritic segregation mechanism. It was shown that in the single-crystalline blades obtained by the directional crystallization using a spiral selector, the “walls” of the primary dendrite arms that grow at a low angle to the blade axis are created. It was found for the first time that the value of the lattice parameter a_γ′_ is decreased near such “walls”. Additionally, it was found that competitive growth of the dendrites may occur at a distance of even several millimeters from the bottom surface of the root. The first-time applied X-ray diffraction measurements of a_γ′_ made in a single-pass along the line allow the analysis of the dendritic segregation in the whole blade cast.

## 1. Introduction

Components of aviation and industrial gas turbines are expected to operate in harsh working conditions, such as high temperature, high pressure, and complex dynamic loading conditions. Hence, the casts of these components are widely produced as single-crystalline using nickel- or cobalt-based superalloys. The most extensively researched and documented in the literature, and nowadays the most frequently applied as well, is the CMSX-4^®^ nickel-based second-generation superalloy. Due to an impressive combination of high-temperature strength, good phase stability, and resistance to oxidation and high-temperature corrosion during operation, the single-crystalline blades made of CMSX-4^®^ superalloy are widely used in a hot section of jet engines [1,2,3,4,5,6,7,8,9].

The single-crystalline casts are commonly produced by directional Bridgman crystallization, during which groups of the γ-phase dendrites are formed. The dendrites form an array with preferred [001]-type crystal orientation consistent with the withdrawal direction of the casting mold from the heating zone [10,11,12,13,14,15]. The single-crystalline superalloys contain several alloying additives that segregate into interdendritic regions or into dendrites on a scale of several hundred microns. The chemical composition heterogeneity related to the dendritic segregation is disadvantageous. Costly complex heat treatment processes are used to reduce heterogeneity that, although largely but not completely, eliminate the effects of dendritic segregation [16,17,18,19]. The residual effects of the segregation may be visualized, for example, in the form of contrast traces in the X-ray topograms obtained for heat-treated samples [20]. Due to complex chemical composition and high amount of refractory elements, in addition to the dominant phases γ and γ′ (over 70%), there are a small fraction of other phases, e.g., topologically close-packed (TCP) phases such as P, R, μ, σ, δ, and the Laves phases C14, C15, C36, which have an adverse effect on the mechanical properties. Their crystal structure consists of close-packed layers of relatively smaller atoms, such as Ni and Cr, between which the larger atoms, such as Mo, W, and Re are located. The TCP phases precipitate in superalloys more often due to long exposure to high temperatures, e.g., due to the heat treatment of constructional parts and during their operation in harsh thermal conditions. The lowest content of these phases has alloys in an as-cast state [21,22,23].

The high-temperature strength of Ni-based superalloys is caused by the formation of the γ′ cuboidal precipitates surrounded by the net of thin channels of the γ matrix. Due to the similar crystal structures and lattice parameters of γ and γ′ phases, the interphase boundaries are coherent, and the material strength is increased by order and coherency strengthening. The coherency strengthening arises from the different lattice parameters of the γ and γ′ phases, imparting strain into the matrix and thereby inhibiting dislocation motion. The lattice misfit between γ and γ′ phases is one of the critical properties that influence the thermal stability and high-temperature mechanical behavior of Ni-based single-crystal superalloys [24,25,26,27]. It is known that changes in the crystal lattice parameters may be related to changes in a lattice misfit and thus also related to changes in strength parameters. It is widely accepted that the large γ/γ′ lattice misfits influence the yield strength, creep life, and rupture life [28,29,30]. The lattice misfit stresses during thermal exposure may influence the dislocation structure [26] and hence—the creation of low-angle boundaries. It follows that the analysis of local changes in the lattice parameter plays an important role in determining possible areas of disadvantageous local changes in mechanical strength.

In the single-crystalline casts of complex shapes, such as turbine blades, the inhomogeneities in the morphology of the dendrite array are formed and hence in their chemical composition. These inhomogeneities occur on a scale of several or a dozen or so micrometers because the growth of the dendrites is controlled by constitutional undercooling related to the diffusion field of alloying elements in the melt ahead crystallization front [1]. The process of forming the structure of the blade cast proceeds in two stages—the crystallization and then the solid-state transformation of the γ-phase. The as-cast γ-dendrite array of the single-crystalline blade obtained by directional crystallization shows heterogeneity in the lattice parameter of the γ-phase. When the temperature is lowered below the solvus temperature, this heterogeneity is inherited by the about one-micrometer size cubes of the γ′-phase formed during the ordering transformation from the primary γ-phase [31]. The structure of single-crystalline blades in a general sense is organized both on a scale of several or even several dozen millimeters, i.e., centimeters scale (the size of the details of entire casts geometry related to the dendrite groups) and on a scale of hundreds of micrometers (the size of the whole dendrite in the transverse section of the blade) as well as on a scale of micrometer (γ′ cube size) and on a scale of nanometers (the order size of the γ′ phase unit cell). These scales of the blade structure are related to the scale-related structural heterogeneities, so the blades’ studies must be proceeded and described on a multi-scale basis.

Highly advanced research methods, such as high-resolution transmission and scanning electron microscopy (HRTEM and SEM), atomic force microscopy (AFM), and scanning tunneling microscopy (STM), allow the structure analysis on a scale from a few nanometers to several hundred nanometers. Still, when using them, it is impossible or very complicated to analyze the macroscopic heterogeneity of the casting structure of the blades resulting from the specificity of their geometry. However, some methods give information about the changes of the nanoscale structure for the points of the macroscopic areas. These include, for example, the positron annihilation spectroscopy (PAS) method [20,32] or the Mössbauer spectroscopy (MS) method [33], as well as X-ray diffraction methods [20,34,35,36,37]. In the PAS and MS methods, data for each measuring point are collected from a sample area of several tens of mm^2^, while the X-ray diffraction methods allow for collecting data from the area above one mm^2^. One of such X-ray methods is the lattice parameter measurement method implemented in the EFG-Freiberg Instruments diffractometer dedicated to analyzing superalloys [38]. The method, which has been used for a short time, allows measuring the lattice parameter of the γ′-phase at points lying on a line with a length of several dozen millimeters. The chosen method of single-pass a_γ′_ measurement allows determining the correlation between a_γ′_ and the dendritic structure on sections of tens of millimeters. HRTEM, SEM, AFM, STM, etc. methods do not allow the measurement of a_γ′_ changes on given sections in single-pass measurement, e.g., for one large sample of the blade.

The analysis of the distribution of a_γ′_ lattice parameter in heat-treated CMSX-4^®^ superalloy is presented in Ref. [39]. Changes in the a_γ′_ related to the dendritic segregation on the scale of one dendrite arm (area below 1 mm^2^) were analyzed using the convergent beam electron diffraction (CBED) method. However, this type of method does not allow to study of such changes in the scale of the entire blade area, that is, in an area with tens of millimeters. Observations of such structure parameters on a nanometric scale are at the cost of limitations in the field of view (the area covered by, e.g., the electron beam). This remark can be applied to several important studies on structure parameters and defects, presented for example in Refs. [40,41,42], the results of which do not allow to conclude about physical phenomena occurring on the scale of the entire casting with a size of tens of millimeters.

For a perfect, dislocation-free model single-crystals, e.g., the single-phase whiskers with a diameter of about 1 mm and a length of about 10 mm, it can be assumed that the structures on the macroscopic scale may be modeled by a simple multiplied unit cell. In contrast, such an approach is not possible for single-crystalline dendritic superalloy blades with a complex shape. In this case, the γ′-phase lattice parameter should be analyzed, considering the structure of single dendrite and dendrite groups. First of all, it is necessary to select and analyze the area of the blade cast where the dendrites grow in a steady-state condition, and their growth is undisturbed by the casting mold walls, as well as the dendritic array probably will be homogeneous. This area will serve as a reference area for analyzing disturbed unsteady dendrite growth areas. The X-ray diffraction result can be most easily interpreted in the reference area. The blade root’s selector extension (SE) area is such an area. The SE area is the cylindrical area bounded by the projection of the selector perimeter into the blade. In the SE area, the dendrites grow in the direction Z_0_ directly from the selector. However, in the h-layer of strictly unsteady dendrite growth [43,44], there is the horizontal transverse growth of the dendrite arms when the crystallization front passes from the selector to the blade root.

The studies were aimed to determine the correlation between the lattice parameter a_γ′_ and the dendritic structure in the single-crystalline model blades made of the nickel-based superalloys by the Bridgman method using a spiral selector. The similar thematically results presented in our previous papers [37,45,46] were related to the macroscopic distribution of a_γ′_ and the dendritic structure studied in different types of as-cast CMSX-4^®^ single-crystalline blades by the X-ray diffraction methods. Changes in a_γ′_(X) relation along the X segments of several tens of millimeters in length allowed to explain some details of the dendritic crystallization processes. However, on the obtained graphs, some local plateaus were observed for the ΔX range from 0.5 to several millimeters, the origin of which has not been previously explained and only assumed that they may be related in some way to the dendritic structure. Confirmation of this assumption and determination the nature of the phenomenon is one of the objectives of the current manuscript, which is the next stage in interpreting results related to the a_γ′_ measurements, dendritic segregation, and dendrite array in the as-cast blades.

## 2. Material and Methods

**Figure 1:**
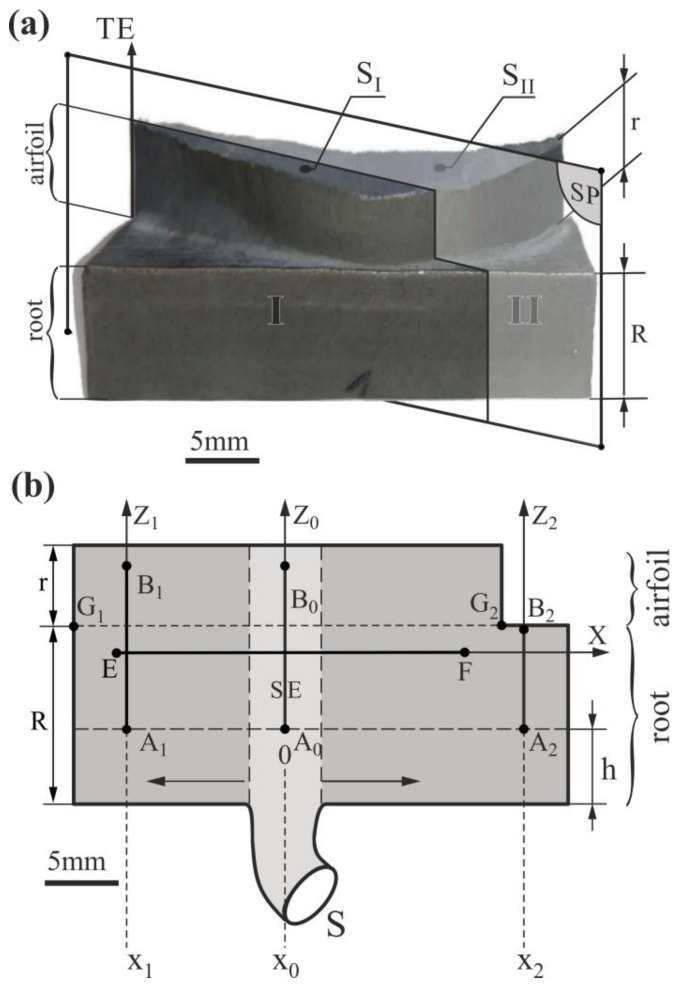
(**a**) The shape of the model blade with marked vertical longitudinal section plane SP dividing the blade into two parts I and II, and fragments S_I_ and S_II_ of top transverse plane of the airfoil, and (**b**) the scheme of the longitudinal surface of part II with marked the a_γ′_ measurement segment E-F of horizontal X-axis line, and segments A_0_–B_0_, A_1_–B_1_, A_2_–B_2_ of vertical Z_0_-, Z_1_-, Z_2_-axes lines. The axes Z_0_, Z_1_, Z_2_ are parallel. TE—trailing edge of the airfoil, SE—selector extension area.

The study model blades were produced in the Research and Development Laboratory for Aerospace Materials, Rzeszów University of Technology, Rzeszów, Poland. The single-crystalline casts made of CMSX-4 superalloy were obtained by the Bridgman directional solidification at the withdrawal rate of 3 mm/min using VIMIC 2 E—DS/SC ALD Vacuum Technologies Co. (Hanau, Germany) vacuum furnace with the vertical temperature gradient G = 16 K/cm. The blades production process was started from preparing wax models, which was used as basis for manufacturing ceramic shell mold. The wax models were obtained by injecting molten wax into a steel matrix. Five models of blades were placed in the wax assemble. Prepared wax assemblies were coated with a ceramic layer through dipping into a refractory slurry and coating with refractory grains followed with drying. Several more layers were applied in a similar way. Total mold thickness was about 9 mm. After the drying process, the wax was removed in steam autoclave and then the mold was fired in at an appropriate temperature and time to remove the remaining wax and to strengthen the ceramic shell. The ingot of CMSX-4^®^ superalloy was inductively melted. The nominal chemical composition of CMSX-4^®^ ingot was as follows (wt %): 5.6 Al, 1.0 Ti, 6.5 Ta, 6.5 Cr, 0.6 Mo, 6.0 W, 9.0 Co, 3.0 Re, 0.1 Hf, less 0.002 C, Ni bal. Melting and solidification processes were carried out in a vacuum. The ceramic shell mold, located on the chill plate, was heated up to 1520 °C and the CMSX-4^®^ melt of the same temperature were poured inside. When the process was finished the mold was removed from the furnace and the blades was knocked out. The spiral selector with channel cross-section diameter d = 5 mm was used for selecting the grain with the orientation of [001]-type crystal direction. The model blades consisted of a bulk root with a length R (Figure 1a) and a relatively small short airfoil with a length r.

The samples for the tests were prepared by intersecting the blade so that the section plane SP (Figure 1a) would pass through the center of the selector extension (SE) area of the root and trailing edge (TE) of the airfoil (Figure 1a). So the blade was divided into parts I and II (Figure 1a). Obtained longitudinal micro-section of part II with the shape presented in Figure 1b was prepared for the tests using the standard for superalloys metallographic procedure [47]. The top transverse surfaces S_I_ and S_II_ were also prepared for the metallographic analysis. The bottom layer h = 5 mm of unsteady growth [43,44] was not analyzed.

The use of a typical commercial alloy such as CMSX-4^®^ and an industrial furnace provided by the ALD Vacuum Technologies applicating proper crystallization parameters, allow obtaining the single-crystalline blades with low (in relation to the γ′-phase) content of the undesirable TCP phases. The share of the γ′-phase, especially taking into account studied longitudinal cross-sections, is large enough to accept the concept of analyzing the correlation between the dendritic structure and lattice parameter, considering the γ′-phase only. The dendritic structure of prepared surfaces’ fragments was observed using scanning electron microscopy (SEM), and the γ′ lattice parameter was measured by the X-ray diffraction method [48] implemented in the Freiberg Instruments EFG diffractometric system (Freiberg Instruments, Freiberg, Germany). A JEOL JMS-6480 scanning electron microscope (JEOL Ltd., Tokyo, Japan) was used to visualize the dendritic microstructure. The images of the analyzed dendritic structure fragments were created by stitching several separate micro-images. The distribution of the a_γ′_ along the three longitudinal vertical lines segments with x_0_, x_1_, x_2_ coordinates, and one transverse horizontal line segment located 2 mm below the border G_1_–G_2_ between the root and the airfoil, was measured. The x_0_ corresponds to the center of the CE area, and x_1_ and x_2_ are 11 mm and 17 mm away, respectively, from x_0_ in opposite directions. The a_γ′_ measurements were performed on the A_0_–B_0_, A_1_–B_1_, A_2_–B_2_, and E–F segments of the measurement lines (Figure 1b). The step between measurement points on the lines was 0.5 mm. The incident beam of characteristic CuKα radiation was 0.8 mm in diameter. The centers of the measurement areas of the a_γ′_ for each point were placed on the measurement lines. The standard error of lattice parameter measurement was 5 × 10^−4^ Å.

## 3. Results and Discussion

**Figure 2:**
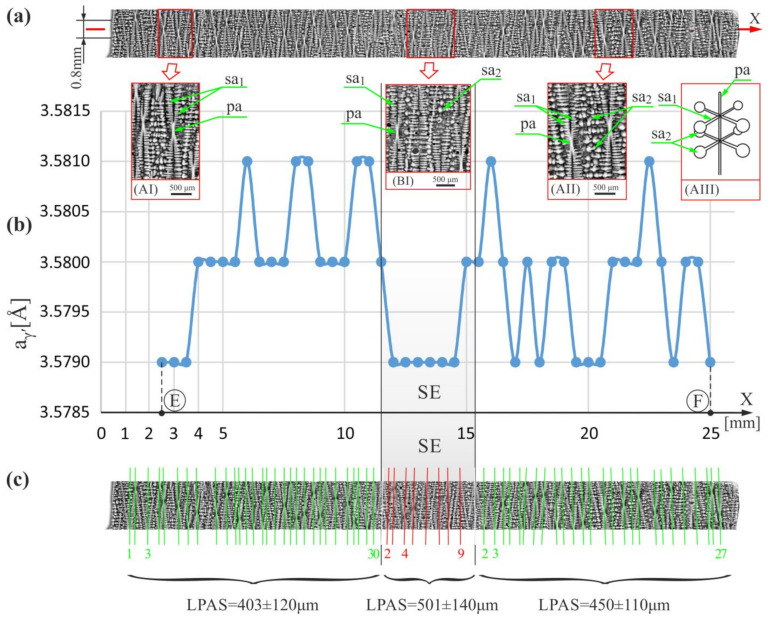
(**a**) The image of the dendritic structure in the measured horizontal strip of micro-section arranged along the X-axis on which the a_γ′_ measurements in the E–F segment were performed, and its enlarged areas AI, AII, and BI, as well as the simplified scheme AIII of a single dendrite, (**b**) the distribution of the a_γ′_ lattice parameter along the X-axis on the E-F segment, and (**c**) the scheme of the primary arms arrangement; E,F—start and end point of the E-F segment, SE—selector extension area.

Figure 2a shows the fragments of the dendritic structure visualized on the longitudinal micro-section of the blade and presented in the form of a horizontal strip corresponding to the segment E–F (Figure 1b) on which the a_γ′_ measurements were performed and in the form of the inserts AI, AII and BI. In the enlarged fragments of the strip (inserts AI, AII, and BI), there are clearly visible images of the dendrites in the form of typical hourglass-like shapes, each of which consists of a vertical segment representing the primary arm (pa) of the dendrite and small, densely arranged horizontal segments representing initial fragments of the secondary arms (sa_1_ in AI, AII, and BI inserts) located near the primary arms (pa). Ending fragments of the secondary arms located at a longer distance from the primary arms are visualized by the teardrop-like shapes that occur between hourglass-like shapes (sa_2_ in AII and AIII inserts).

Figure 2b shows the distribution of the a_γ′_ parameter, measured along the X-axis in the strip area of 0.8 mm wide related to the primary X-ray beam (Figure 1b). From the graph analysis, it can be concluded that the values of the a_γ′_ inside the whole strip change in the range from 3.5790 to 3.5810 Å. In contrast, in the SE area, the values of the a_γ′_ have a lower range of changes—from 3.579 to 3.5800 Å. Therefore it can be concluded that the mean value of the a_γ′_ in the SE area is less than that measured in the other root areas.

Figure 2c shows the scheme of the arrangement of the primary dendrite arms presented in Figure 2a, which are represented by almost vertical segments parallel to the axes of the hourglass-like shapes. The distances between the segments representing so-called linear primary arm spacing (LPAS) were measured using the method described in Refs. [43,45]. The measurements with this method were performed using the scheme (Figure 2c) prepared by creating skeletal images. Skeletonization is a technique whereby a binary image of dendrites is eroded step by step until the skeleton of the image is obtained. The skeletal image is created as a thin line equidistant from the original edges of the binary dendrites’ shape. The LPAS is the average distance between the primary arms shape of the dendrites visualized on the longitudinal surface section and measured along one straight line. The spaces were determined by digital image processing measurements. The relatively large mean errors in determining the LPAS were related to the small number of dendrites in each area. However, it should be noted that an LPAS analysis is especially useful for visualizing the changes in interdendritic distances of the primary arms in the longitudinal section and for comparing the difference in the LPAS values between neighboring areas. The LPAS value in the SE area was 501 ± 140 μm for the 10 dendrite arms, in the strip area to the left of the SE—it was 403 ± 120 μm for the 31 dendrite arms, and in the strip area to the right of the SE—it was 450 ± 110 μm for the 27 dendrite arms. It can be concluded that in the SE area, the distances between primary dendrite arms are greater than in the other areas of the strip, which can be explained as follows. With the passing of the crystallization front from the selector to the root, in the h-layer outside the SE area, the rapid lateral growth of the secondary dendrite arms (the horizontal arrow—Figure 1b) exists [43]. The densely arranged tertiary arms grow vertically in unsteady conditions from the secondary arms. Above the h-layer, these arms can be called primary arms (pa) for simplicity. In the SE area of the h-layer and above, the primary arms that grow vertically directly from the selector in steady conditions are longer distant from each other, and the LPAS value is higher. Of course, above the h-layer, as the growth process outside the SE area begins steadied, the LPAS value in all areas outside the SE should gradually equalize to the LPAS of the SE area by the competitive growth mechanism. However, for the analyzed model blades, which have a relatively small root height, the equalization process at the E–F strip level did not have time to end. The incompleteness of stabilizing the growth process in the areas outside the SE is related to the incompleteness of the competitive dendrites growth and may cause higher a_γ′_ fluctuations visible in Figure 2b compared to the SE area.

**Figure 3:**
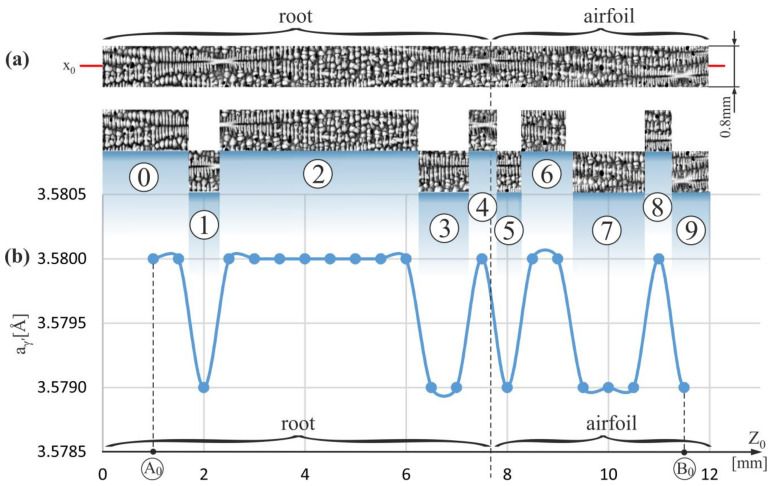
(**a**) The image of the dendritic structure in the measured vertical strip of micro-section with the coordinate x_0_ along which the a_γ′_ measurements were performed, and (**b**) the distribution of the a_γ′_ lattice parameter along Z_0_-axis on the A_0_–B_0_ segment (Figure 1b); the numbers 0–9 indicate the strip fragments.

Figure 3a shows a fragment of the dendritic structure visualized on the longitudinal micro-section of the blade root. The fragment is presented as a strip of the A_0_–B_0_ segment (Figure 1b) with the coordinate x_0_, where the a_γ′_ measurements were performed. The results of the measurements are presented in Figure 3b. The a_γ′_ parameter was measured in the strip area of 0.8 mm wide related to the primary X-ray beam. Below the dendritic structure of the strip, its fragments 1–4 are presented, which correspond to the areas of increased or decreased the a_γ′_ value evident in Figure 3b. It can be noted that the a_γ′_ decrease occurs in the areas of the root, where parallel, densely arranged initial fragments of the secondary dendrite arms predominate (fragments 1 and 3). On the other hand, the increase of the a_γ′_ value occurs in the root areas, where the ending fragments of the secondary dendrite arms (fragments 2 and 4), visible as the teardrop-like shapes, predominate. For some areas of the fragments 1–4, this type of correlation is less pronounced because the X-ray beam covered an area from the a_γ′_ measurement data was collected, with a diameter slightly larger than the distance of 0.5 mm between neighboring measurement points. The correlation is more noticeable when more structure fragments are analyzed simultaneously, e.g., by comparing fragments 1–4.

The growth of the dendrites and dendritic segregation of alloying additives in a thin-walled airfoil are strictly non-equilibrium (unsteady conditions) because the geometry of a typical airfoil (with inclined and twisted surfaces of the mold) strongly affect the processes mentioned above. For this reason, the correlation between a_γ′_ and the dendritic structure can be very complex and difficult to observe. Dendritic segregation in bulk root and especially in the SE area of the root is largely devoid of the influence of mold walls. Therefore, it should be assumed that dendritic crystallization in the SE area of the root is almost equilibrium (steady), and observation of a correlation between a_γ′_ and dendritic structure can be simplified.

A similar but less evident correlation between the a_γ′_ values and the structure of the dendrites is noticeable in the airfoil area of the strip. It can be related to the disturbance of the steady growth process of the dendrites in the airfoil, where the frequency of the dendrites’ interaction with the casting mold walls increases. This type of disturbances may also occur for fragment 0, located on the border with the area of strictly unsteady dendrites growth (h-layer, Figure 1b).

**Figure 4:**
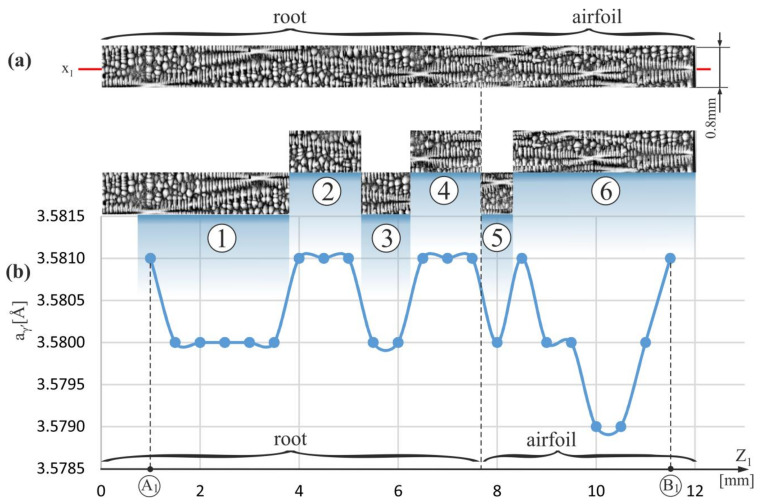
(**a**) The image of the dendritic structure in the measured vertical strip of micro-section with the coordinate x_1_ along which the a_γ′_ measurements were performed, and (**b**) the distribution of the a_γ′_ lattice parameter along Z_1_-axis on the A_1_–B_1_ segment (Figure 1b); the numbers 1–6 indicate the strip fragments.

**Figure 5:**
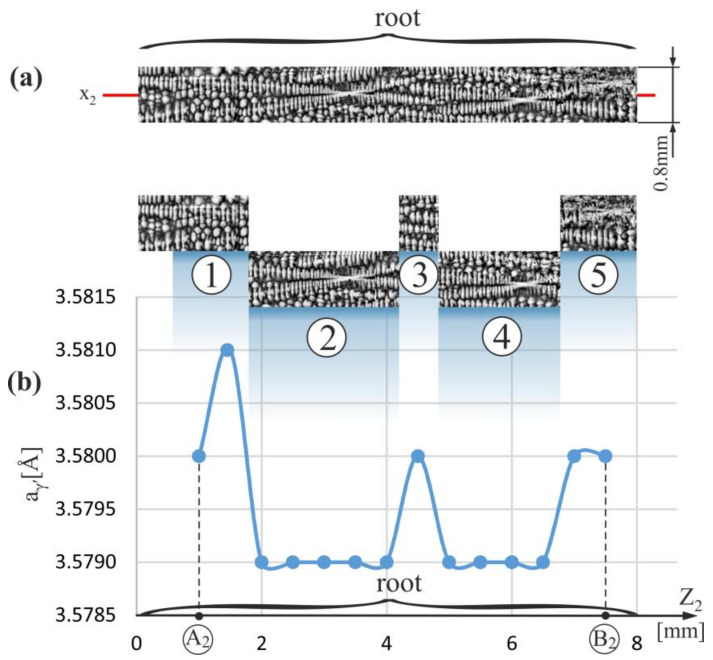
(**a**) The image of the dendritic structure in the measured vertical strip of micro-section with the coordinate x_2_ along which the a_γ′_ measurements were performed, and (**b**) the distribution of the a_γ′_ lattice parameter along Z_2_-axis on the A_2_–B_2_ segment (Figure 1b); the numbers 1–5 indicate the strip fragments.

A similar correlation can be observed by analyzing Figure 4 and Figure 5, which show the dendritic structure and the distribution of the a_γ′_ parameter for strips of the A_1_–B_1_ and A_2_–B_2_ segments located outside the SE area. In these strips, the conditions of the dendrites growth may not yet be fully steadied (are quasi-stable).

An increase in the range of the a_γ′_ changes and a disturbance of the above-described correlation is observed for points of a strip of the A_1_-B_1_ segment in the airfoil. A similar effect is observed for the strip of the A_2_-B_2_ segment near the h-layer (near A_2_ point—Figure 5). Although for most fragments of the root such above-described correlation between the dendritic structure and the a_γ′_ value is clearly visible (fragments 1–4 on Figure 3), however, already in the airfoil fragments and in the root fragments located near the airfoil and/or near the h-layer of strictly unsteady growth (near the points A_0_, A_1_, A_2_), there is a disturbance of this correlation and/or an increase in the range of the a_γ′_ changes.

Thus, it can be concluded that the above-described correlation between the dendritic structure and changes in the a_γ′_ parameter is most characteristic for the area of strictly steady growth of the dendrites, which for the studied blades occurs inside the SE area of the root. The reason for this type of correlation may be dendritic segregation. It is known that as a result of dendritic segregation, Al, Ti, Ta segregate into interdendritic areas increasing the a_γ′_ value [46]. The ending fragments of the secondary dendrite arms, which are visible on the micro-section as the teardrop-like shapes, crystallize from the residual interdendritic liquid in which an already increased amount of Al, Ti, Ta occurs resulting from earlier stages of the crystallization of the initial fragments of these arms. For this reason, in the areas with a higher share of the ending fragments of the secondary arms, the already increased concentration of Al, Ti, Ta will be further increased, which will increase the a_γ′_ value.

Deviation from the conditions of the steady growth of the dendrites in the root may take place not only in the h-layer of strictly unsteady growth or in its vicinity and in the airfoil, but also in certain fragments of the casts where the higher density of the spatial distribution of the dendrites is observed. Such area is visible in fragment 6 of the dendritic structure presented in Figure 4.

**Figure 6:**
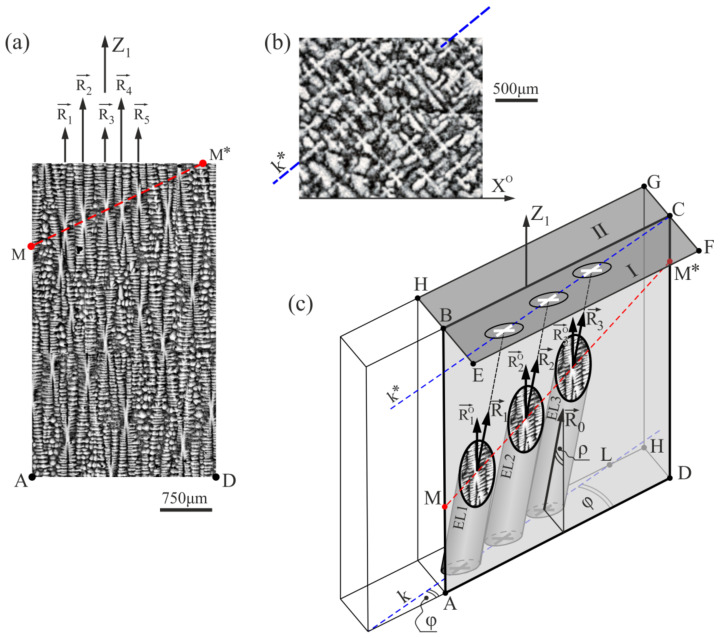
(**a**) The image of the dendritic structure visualized on the part of the longitudinal section containing fragment 6 presented in Figure 4; (**b**) the image of the dendritic structure visualized on the transverse surface S_I_ (Figure 1a) near the fragment 6, and (**c**) the scheme of the dendrites set cut by the section plane ABCD. The Z_1_-axis is parallel to the Z_0_-axis presented in Figure 1b; EL1–EL3— the outline of the primary dendrites.

Figure 6a shows the dendritic structure of the area extended around fragment 6 shown in Figure 4. It presents a set of five dendrites, visualized by the vectors R_1_–R_5_, arranged closer to each other in relation to the other dendrites visible on the micro-section. Additionally, the primary dendrite arms images (hourglasses-like shapes center) are arranged on a straight line MM*. The dendritic structure of this area, visualized on the transverse surface of the blade airfoil (surface S_I_, Figure 1a) and shown in Figure 6b, indicates the presence of an image of the dendrites chain along the line k*. These types of chains are often observed in a root’s h-layer of strictly unsteady growth [43]. The dendrites, creating the chain, form some kind of the dendritic “walls” inclined by a small angle to the Z_1_-axis (Figure 1b). The scheme of creating an image of one of such “walls” on the longitudinal ABCD section is presented in Figure 6c. In the h-layer of the root, a chain of k-type is formed from one lateral so-called leading arm [44], from which the arms R_1_-R_5_ grow in the direction Z_1_ and create a dendritic wall. Such arms on the transverse section of the root are usually called the primary. The mechanism of chain formation is described in detail in Ref. [43]. The wall formed by the primary arms, three of which are shown in Figure 6c, grows inclined at a small angle ρ relative to the surface of the longitudinal micro-section. It is also rotated by the φ angle about the horizontal AD edge of the micro-section. As a result of the dendritic wall’s inclination and rotation on the micro-section’s vertical longitudinal plane, a set of hourglass-like shapes arranged on the line MM* is visible. A chain of dendrite images of a four-petal flower morphology positioned on the line k* (Figure 6) is also visible on the horizontal transverse surface of the upper micro-section. Such local concentration of the primary dendrites arms results in a concentration of the initial fragments of the secondary arms and a higher reduction of the parameter a_γ′_ (Figure 4, fragment 6 near the coordinate z_1_ = 10). A similar effect is visible in Figure 2b in the area with the coordinates of x = 1, 2, 3, 4, for which concentration of the ending fragments of the secondary dendrite arms is observed. This phenomenon is unfavorable because it causes both the heterogeneities of the dendrite structure and the chemical composition of the cast. Of course, when the blade is slim (the dimension in the Z_1_ direction is significantly larger than in perpendicular directions), these types of “walls” can fade in the long root or long airfoil due to stochastic fluctuations.

The mean spacing of the primary dendrites arms in the root’s SE area at the distance of several millimeters from the bottom surface of the root is higher than the spacing outside the SE area. It means that for blades with a geometry characterized by a relatively short root (with a length of several millimeters), the mechanism of the competitive growth does not keep up to stabilize the spacing between the primary arms outside SE to the equilibrium value characteristic of a given crystallization rate and temperature gradient.

In some areas of the blade, the local disturbances in the dendrites growth can be caused by the “walls” of primary dendrites arms formed during the transition of the crystallization front from the selector to the root and their propagation in the almost vertical direction to the other blade fragments. In such areas, the lattice parameter a_γ′_ decreases by about 0.002 Å compared to the a_γ′_ value in subareas where the ending fragments of the secondary arms are predominant.

## 4. Conclusions

In the root, especially in its selector extension (SE) area, there are subareas in which the initial fragments of the secondary dendrite arms predominate, and the dendritic segregation causes a decrease in the lattice parameter of the γ′-phase (a_γ′_). The value of this decrease is of the order of 0.001Å compared to the a_γ′_ value in other subareas where ending fragments of the secondary dendrite arms are predominant. The initial fragments of the secondary arms are visualized on the longitudinal section by the hourglass-like shapes and their ending fragments by the teardrop-like shapes.

In the areas of the blade with local disturbances in the dendrites growth, the lattice parameter a_γ′_ decreases by about 0.002 Å compared to the a_γ′_ value in subareas where the ending fragments of the secondary arms are predominant.

The mean spacing of the primary dendrites arms in the root’s SE area at the distance of several millimeters from the bottom surface of the root is higher than the spacing outside the SE area.

The first-time applied X-ray diffraction measurements of a_γ′_ made in a single-pass along the line allow the analysis of the dendritic segregation in the whole blade cast. It is not possible with other methods, e.g., highly advanced techniques of SEM, TEM, AFM, STM, etc., that analyze data from micro- and nano-areas.
